# Effects of a Multimodal Neurorehabilitation Program on Gross Motor Function and Activity and Participation Performance in Children With Cerebral Palsy: A 9‐Month Longitudinal Study

**DOI:** 10.1002/pri.70158

**Published:** 2025-12-22

**Authors:** Franciele Zardo, Daiane Bridi, Monique Schorn, Isabelle Cousseau, Ketlyn Leal, Sabrina Knop, Fernanda Cechetti

**Affiliations:** ^1^ Postgraduate Program in Rehabilitation Sciences Federal University of Health Sciences of Porto Alegre (UFCSPA) Porto Alegre Rio Grande do Sul Brazil; ^2^ Federal University of Rio Grande do Sul (UFRGS) Porto Alegre Rio Grande do Sul Brazil; ^3^ Feevale University Novo Hamburgo Rio Grande do Sul Brazil; ^4^ Anhanguera College Caxias do Sul Rio Grande do Sul Brazil; ^5^ Department of Physiotherapy and Researcher in the Postgraduate Program in Rehabilitation Sciences Federal University of Health Sciences of Porto Alegre (UFCSPA) Porto Alegre Rio Grande do Sul Brazil

**Keywords:** cerebral palsy, longitudinal studies, motor activity, neurological physiotherapy, patient participation

## Abstract

**Introduction:**

Long‐term physiotherapy is essential to enhance motor and functional development in children with cerebral palsy (CP). The aim of this study was to analyze the effects of multimodal neurorehabilitation in children with CP by evaluating its effectiveness over time at all levels of the Gross Motor Function Classification System (GMFCS) on gross motor function, activity and participation.

**Methods:**

This prospective, longitudinal cohort study aimed to characterize a sample and evaluate the effectiveness of multimodal neurorehabilitation over a 9‐month period. Gross motor function was assessed using the Gross Motor Function Measure (GMFM‐66), and performance in activity and participation was assessed using the Pediatric Evaluation of Disability Inventory (PEDI). The assessments were performed at four assessment points.

**Results:**

29 children diagnosed with CP aged 11 months to 12 years and from all GMFCS levels were included in the study. Neurorehabilitation improved gross motor function in both groups in the total score. Groups IV and V improved only in dimension B (sitting), while groups I, II and III showed significant changes in dimensions D (standing) and E (walking, running and jumping). Regarding mobility and social function, only levels I, II and III showed significant differences.

**Conclusion:**

These findings reinforce that a multimodal neurorehabilitation program, when structured within a standardized clinical protocol and tailored to the functional profile of each GMFCS level, may contribute meaningfully to motor development in children with CP. Furthermore, it promoted advances in mobility and social function at levels I, II, and III. At the more severe levels (IV and V), specific improvements in sitting posture were observed.

## 1 Introduction

Long‐term physiotherapy is essential for children with Cerebral Palsy (CP) given the complexity and heterogeneity of the clinical manifestations of this condition. The definition of cerebral palsy proposed by Rosenbaum et al. (2007) characterizes the condition as a group of permanent developmental disorders affecting movement and posture, leading to activity limitations and resulting from non‐progressive disturbances in the developing fetal or infant brain. These motor impairments are commonly accompanied by alterations in sensation, perception, cognition, communication, behavior, epilepsy, and secondary musculoskeletal complications. CP is the most prevalent physical disability in childhood and has lifelong impact, influencing not only motor performance but also social participation, autonomy, and overall quality of life (Novak et al. 2013; Saleh et al. 2019). Therefore, it is important to investigate interventions that promote neuroplasticity, functionality and social inclusion, ensuring that these children reach their maximum potential (Colver et al. 2014).

Rehabilitation centers for CP should adopt evidence‐based approaches that consider the individual characteristics of each child, such as level of functional capacity, comorbidities, age and environmental context (Colver et al. 2014). Among the most promising strategies are interventions based on the principles of neuroplasticity, which include active task‐specific movements performed at high intensity and with environmental modifications. However, guidelines for clinical practice are still limited due to small sample sizes and variability of interventions, treatment duration, age groups and outcomes assessed (Morgan et al. 2016; Novak et al. 2020). Evidence remains limited, especially in longitudinal research investigating impacts on different approaches and their relationship with gross motor function, activity and participation (Berker and Yalçin 2008; Jackman et al. 2022; Chagas et al. 2023; Lidbeck et al. 2023).

Recent studies highlight the importance of interventions that involve the family in the therapeutic process and consider the multiple factors that influence the child's development, such as environmental support, accessibility and motivation (Morgan et al. 2016; Novak et al. 2020). However, there are still gaps regarding the effectiveness of integrated and longitudinally monitored approaches. Among the most used interventions with this population are the Neurodevelopmental Treatment (NDT), locomotor training and suit therapies. NDT emphasizes individualized therapeutic management, based on movement analysis for habilitation and rehabilitation of individuals with neurological pathophysiology (Neuro‐Developmental Treatment Association (NDTA) Instructors Group, 2016; Kollen et al. 2009; Furtado et al. 2022). Treadmill locomotor training can improve mobility and be an effective complement to overground walking (Jackman et al. 2022) and the suit therapies have been gaining popularity but still lack robust validation (Novak et al. 2020; Furtado et al. 2022).

Previous studies have analyzed specific neurorehabilitation approaches applied under standardized protocols; however, few have examined multimodal combinations of techniques within integrated programs (Neves et al. 2013; Camara Machado et al. 2017; Da Silva Júnior 2018; Raipure et al. 2023). Others follow children for a period of time to investigate correlations, such as gross motor skills and mobility (Burgess et al. 2022), mobility and self‐care activity (Kim et al. 2017), the development of a child with spastic hemiparesis presenting predominant involvement in one body side (Eliasson et al. 2023), or describe characteristics, functioning and deficiencies as in the large multicenter cohort study in Brazil (PartiCipa Brasil) (Chagas et al. 2024). However, there are few studies investigating the combined effectiveness of integrated and longitudinally monitored programs on different components of the ICF.

Given this scenario, the present study aims to understand the effects of neurorehabilitation in children with CP by evaluating its effectiveness over time at all levels of the Gross Motor Function Classification System (GMFCS). Through longitudinal monitoring, we seek to investigate the impacts of the multimodal neurorehabilitation program on gross motor function, activity and participation. This understanding is essential to support more effective, integrated and personalized clinical practices.

## 2 Methods

This was a prospective, longitudinal cohort study approved by the Ethics Committee of the Universidade Federal de Ciências da Saúde de Porto Alegre (No. 5.040.891; CAAE 50820221.9.0000.5345). ClinicalTrials.gov registration ID NCT05608954 XXXXXXXX.

### 2.1 Participants

Inclusion criteria were diagnosis of CP established by a pediatric neurologist following Rosenbaum et al. (2007) criteria; confirmation via clinical exam and developmental history; inclusion of infants from 11 months to 12 years; and at all levels of the GMFCS. Exclusion criteria were application of botulinum toxin 6 months before intervention, minimum interval of 6 months between orthopedic surgery and intervention onset and presenting other physical abnormalities, genetic or severe syndromes. Written informed consent was obtained from the children's guardians before the start of this study.

### 2.2 Assessment Procedures and Instruments

The study was carried out between February and November 2023 at neurorehabilitation centers located in Rio Grande do Sul, Brazil. Data were collected at four assessment points, referred to as “time T”: February (initial assessment—T0), May (first reassessment—T1), August (second reassessment—T2) and November (last reassessment—T3). All assessments were conducted by the same researchers.

Participants were assessed using the following instruments: (a) Initial Assessment Form: participant identification data and information relevant to the research, such as spasticity classification using the Modified Ashworth Scale, which ranges from 0 (no increase in tone) to 4 (severe rigidity) (Meseguer‐Henarejos et al. 2018; Harb and Kishner 2023) and the assessment of the respiratory system such as chest expansion and presence/absence of retraction; (b) Questionnaire for Parents and/or Caregivers: information on caregivers, child activities, family psychological support, age of diagnosis, start age of therapies, therapy frequency and modalities, school type, use of walking aids/orthoses, and daily assistance level (dependent/independent); (c) Therapy Control Spreadsheet: records of the frequency and duration of sessions, methods used and attendance during the research period, completed by the therapist responsible for the child. All these instruments were developed by the main researcher.

The instrument used for screening was the Gross Motor Function Classification System (GMFCS), a functional classification system composed of five levels, used to assess the motor skills of children with CP. The levels are I: walks without limitations, II: walks with limitations, III: walks with the aid of a manual mobility device, IV: has limited mobility and can use a motorized chair and V: needs to be transported in a manual wheelchair (Ho et al. 2017). This classification facilitates the identification of specific needs and the comparison of treatment results (Palisano et al. 1997). Children were grouped into two large groups based on their functional level: The first group included individuals at levels I, II, and III, representing the least impaired. The second group included individuals at levels IV and V, representing the most impaired.

To measure the impact of the neurorehabilitation, we used the Gross Motor Function Measure (GMFM‐66) and the Pediatric Evaluation of Disability Inventory (PEDI), applied at times T0, T1, T2 and T3 and T0 and T3, respectively. Both tests were applied by the same researchers who followed standardized procedures.

GMFM‐66 is an observational scale used to measure gross motor function in children with CP. It consists of 66 items divided into dimensions that assess skills such as lying down, rolling over, sitting, crawling, kneeling, standing, walking, running, and jumping. The score ranges from 0 (no skill) to 3 (maximum skill), resulting in a final score ranging from 0 to 100 (Russell et al. 2000; Ho et al. 2017). The software Gross Motor Ability Estimator (GMAE‐2) (CanChild, Hamilton, ON, Canadá) was used to calculate GMFM‐66 scores.

PEDI assesses children's functional abilities in activities of daily living through two sections: Part I—Functional Skills: 197 items that verify self‐care, mobility and social function, with binary scoring (0 = incapable; 1 = capable). Part II—Caregiver Assistance: 20 items to assess the level of support needed during activities. Each domain generates three scores: level of functional ability, caregiver assistance and necessary modifications. Higher scores indicate greater performance and independence (Feldman et al. 1990; De Mello Sposito and Riberto 2010; Haley et al. 2010). In this study, the total score for each area was calculated based on the normative score for children aged 6 months to 7 years and 11 months and the continuous score for those older than 7 years and 11 months, with the aim of analyzing the difference between the beginning and the end of the study.

During the study, participants maintained their usual routines and therapies to measure the real benefits of the therapies they were already used to. Approximately 3 weekly sessions were held at 3 neurorehabilitation centers.

The methods used in the rehabilitation sessions included NDT, suit therapies, locomotor training on a treadmill and in the community, elastic bandage, psychomotricity and support from multidisciplinary areas (speech therapy, psychopedagogy and occupational therapy). The physiotherapy sessions were carried out with the therapists who already treated these children. All therapists involved held NDT Level‐1 certification and completed 20 h of study‐specific training, with biweekly fidelity checks and were certified in GMFM scoring.

### 2.3 Statistical Analysis

The results of the qualitative variables were presented as absolute and relative frequencies and the quantitative variables as mean and standard deviation. Normality was verified by the Shapiro–Wilk test. The Chi‐Square test, Fisher's exact test and Student's *t*‐test were used to compare the variables according to GMFCS. To verify the change in GMFM‐66 and PEDI in each group, the student's *t*‐test for paired data and ANOVA of repeated measures with Sidak's test for multiple comparisons were used. The significance level adopted was 0.05 and the analyses were performed using the statistical software SPSS (IBM SPSS Statistics for Windows, Version 25.0. Armonk, NY: IBM Corp.).

## 3 Results

Sixty‐five children with CP were invited to participate in the study and 29 were included in a convenience sample. Three participants were lost, one due to a change of neurorehabilitation center, one due to the need for surgery during the study, and one due to death. The sample characteristics are described in Table 1 and Standardized Intervention Protocol in Table 2. Despite the differences between the groups, our sample showed homogeneity in most of the variables analyzed. However, we observed that the hamstring muscles were more spastic in groups IV and V (62.5%), most of the children used walking aids (75%) and were totally dependent (87.5%), and 100% had quadriparesis.

**TABLE 1:** Sample characterization according to GMFCS.

		Total (*n* = 29)		I, II and III (*n* = 21)		IV and V (*n* = 8)		*p* value
		*n*	%	*n*	%	*n*	%	
Age	mean ± SD	6.9 ± 3.4		6.9 ± 3.3		6.9 ± 3.9		0.967
	0–6 years	12	41.4	10	47.6	2	25.0	0.408
	7–12 years old	17	58.6	11	52.4	6	75.0	
Age at diagnosis (months) (*n* = 26)	mean ± SD	8.2 ± 7.4		7.7 ± 6.7		9.4 ± 9.1		0.608
Treatment start (*n* = 25)	Before diagnosis	4	16.0	2	11.8	2	25.0	0.570
	After diagnosis	21	84.0	15	88.2	6	75.0	
Gender	F	13	44.8	10	47.6	3	37.5	0.697
	M	16	55.2	11	52.4	5	62.5	
Support network (*n* = 26)	Parents with help	9	34.6	7	38.9	2	25.0	0.667
	Only father and/or mother	17	65.4	11	61.1	6	75.0	
Child participates in activities (*n* = 26)	Yes	23	88.5	17	94.4	6	75.0	0.215
	No	3	11.5	1	5.6	2	25.0	
Psychological support for caregivers (*n* = 26)	Yes	5	19.2	3	16.7	2	25.0	0.628
	No	21	80.8	15	83.3	6	75.0	
Most spastic muscle(s)	Adductor	3	10.3	1	8.3	2	25.0	0.176
	Quadriceps	3	10.3	1	8.3	2	25.0	0.176
	Biceps	1	3.4	1	8.3	0	0.0	1000
	Hamstrings	8	27.6	3	25.0	5	62.5	**0.019** ^*^
	Plantar flexors	13	44.8	10	83.3	3	37.5	0.697
	Wrist flexors	5	17.2	3	25.0	2	25.0	0.597
	Gastrocnemius	5	17.2	4	33.3	1	12.5	1000
Attends school (*n* = 28)	Yes	25	89.3	19	95.0	6	75.0	0.188
	No	3	10.7	1	5.0	2	25.0	
School type	Regular	23	92.0	17	89.5	6	100.0	1000
	Specialized	2	8.0	2	10.5	0	0.0	
Uses an assistive device for walking (*n* = 28)	Yes	10	35.7	4	20.0	6	75.0	**0.011** ^*^
	No	18	64.3	16	80.0	2	25.0	
Uses orthosis (*n* = 28)	Yes	17	60.7	11	55.0	6	75.0	0.419
	No	11	39.3	9	45.0	2	25.0	
Dependency level (*n* = 27)	Total	10	37.0	3	15.8	7	87.5	**0.002** ^*^
	Partial	12	44.4	11	57.9	1	12.5	
	Independence	5	18.5	5	26.3	0	0.0	
Therapies (*n* = 28)	Bobath	28	100.0	20	100.0	8	100.0	IN
	Locomotor training	14	50.0	9	45.0	5	62.5	0.678
	Psychomotricity	10	35.7	8	40.0	2	25.0	0.669
	RTA	6	21.4	6	30.0	0	0.0	0.141
	Pediasuit	2	7.1	1	5.0	1	12.5	0.497
	Others	15	53.6	12	60.0	3	37.5	0.410
Duration of therapies (*n* = 28)	At least 1 h	9	32.1	7	35.0	2	25.0	1000
	< 1 h	19	67.9	13	65.0	6	75.0	
Weekly frequency (*n* = 28)	mean ± SD	2.6 ± 1.2		2.5 ± 1.3		3.0 ± 0.9		0.326
MAS	1	5	17.2	5	23.8	0	0.0	0.265
	1+	4	13.8	4	19.0	0	0.0	
	2	8	27.6	5	23.8	3	37.5	
	3	8	27.6	5	23.8	3	37.5	
	4	4	13.8	2	9.5	2	25.0	
Topography	Hemiparesis	10	34.5	10	47.6	0	0.0	**<** **0.001** ^*^
	Quadriparesis	11	37.9	3	14.3	8	100.0	
	Diparesis	7	24.1	7	33.3	0	0.0	
	Hypotonia	1	3.4	1	4.8	0	0.0	

*Note:* Data presented in %, mean ± SD (mean and standard deviation).

Abbreviation: RTA: thoracoabdominal rebalancing.

^*^ *p*‐value < 0.05.

**TABLE 2:** Standardized intervention protocol.

Intervention	Operational parameters	Progression rules	Modification/suspension criteria
Neurodevelopmental treatment (NDT/Bobath) (all children)	40–60 min, 1–5 ×/week; focus on postural control, transitions, proximal alignment.	Increased postural challenge when the child maintains control ≥ 70% of the session; introduction of more complex tasks as tolerated.	Excessive fatigue, pain, or loss of significant postural control.
Treadmill locomotor training	Initial speed 0.8–1.0 m/s; max 15 min, 2–3 ×/week.	Speed increased by 0.1 m/s after two consecutive sessions maintaining ≥ 75% gait symmetry.	Postural instability, pain, or decreased cardiovascular performance.
Pediasuit/intensive suit‐based therapy	3–4 h, 5 ×/week; resisted functional exercises and alignment training.	Gradual increase in elastic resistance based on functional performance.	Skin irritation, severe fatigue, persistent refusal.
Psychomotricity	40–60 min, 1–2 ×/week; focus on body schema, coordination, and motor exploration.	Introduction of more complex tasks as motor organization improves.	Excessive agitation or emotional discomfort.
Functional taping	Weekly applications; techniques for postural alignment and motor facilitation.	Weekly adjustment based on muscular/postural response.	Skin irritation.
Complementary therapies (speech therapy, educational psychology, occupational therapy, early stimulation)	Frequency 1–2 ×/week according to interdisciplinary plan.	Conducted according to professional protocols.	—

**FIGURE 1:**
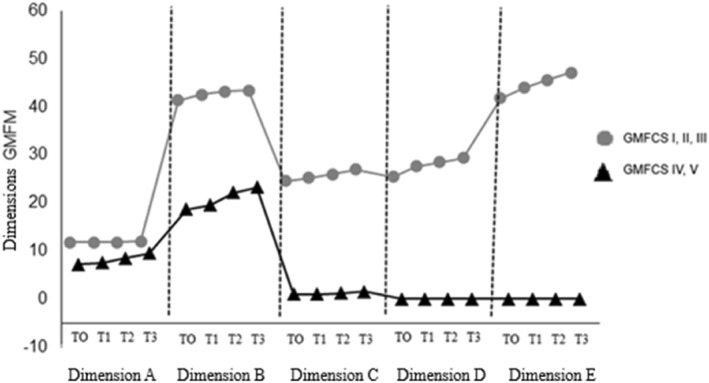
Relationship between GMFM dimensions according to GMFCS levels. GMFM, gross motor function measurement. Dimension A, lying down and rolling; B, sitting; C, crawling and kneeling; D, standing; E, walking, running and jumping. T0 (assessment), T1 (1st reassessment), T2 (2nd reassessment) and T3 (last reassessment) in each dimension.

The relationship between GMFM‐66 score and the GMFCS levels is presented in Table 2. The analysis of gross motor function by GMFM‐66 dimension—A (lying down and rolling over), B (sitting), C (crawling and kneeling), D (standing) and E (walking, running and jumping) was also performed for each GMFCS level and described in Table 3 and Figure 1, considering the same assessment moments.

**TABLE 3:** Relationship between gross motor function total score and in each dimension (GMFM) according to GMFCS.

	GMFCS	T0	T1	T2	T3	*p* value
GMFM total score	I, II and III	66.1 ± 19.1a	69.5 ± 19.5b	72.2 ± 20.3bc	73.7 ± 19.8c	**<** **0.001** ^*^
	IV and V	28.6 ± 10.1a	29.5 ± 9.9ab	30.7 ± 9.1b	31.9 ± 8.3b	**<** **0.001** ^*^
Dimension A	I, II and III	11.8 ± 0.9	11.8 ± 0.7	11.8 ± 0.7	12 ± 0	0.4
	IV and V	7.3 ± 4.9	7.6 ± 4.9	8.6 ± 3.9	9.6 ± 3.3	0.079
Dimension B	I, II and III	41.3 ± 6.7	42.6 ± 4.9	43.2 ± 3.7	43.4 ± 3.7	0.071
	IV and V	18.7 ± 11.4a	19.6 ± 11.1ab	22.1 ± 13.1ab	23.3 ± 12.8b	**<** **0.001** ^*^
Dimension C	I, II and III	24.6 ± 9.1	25.3 ± 8.8	26 ± 7.4	27 ± 7	0.069
	IV and V	1 ± 2.6	1 ± 2.6	1.3 ± 2.6	1.6 ± 2.8	0.415
Dimension D	I, II and III	25.5 ± 12.3a	27.6 ± 12.3ab	28.5 ± 12.5b	29.3 ± 12.3b	**<** **0.001** ^*^
	IV and V	0 ± 0	0 ± 0	0 ± 0	0 ± 0	IN
Dimension E	I, II and III	41.8 ± 26.6a	44 ± 26.9a	45.6 ± 26.9b	47.1 ± 25.9b	**<** **0.001** ^*^
	IV and V	0 ± 0	0 ± 0	0 ± 0	0 ± 0	IN

*Note:* Data are presented as mean ± SD (mean and standard deviation). Means that do not share letters differ from each other. Sidak test for multiple comparisons.

Abbreviations: GMFCS, gross motor function classification system; GMFM, gross motor function measure; NA, not evaluated.

^*^ *p*‐value < 0.05.

In the overall GMFM‐66 score, T3 presented higher values when compared to T0 and T1, while T1 and T2 were higher than the T0 for levels I, II and III (*p* < 0.001). For levels IV and V, the score at T3 was significantly higher than T0, T1 and T2 (*p* < 0.001). In the stratification, groups IV and V showed significant improvement only in dimension B (sitting) at T3 in relation to T0 (*p* < 0.001). The group less impaired demonstrated progressive improvements, with scores at T1, T2 and T3 higher than T0 (*p* < 0.001) in dimension D (standing) and in dimension E (walking, running and jumping).

**FIGURE 2:**
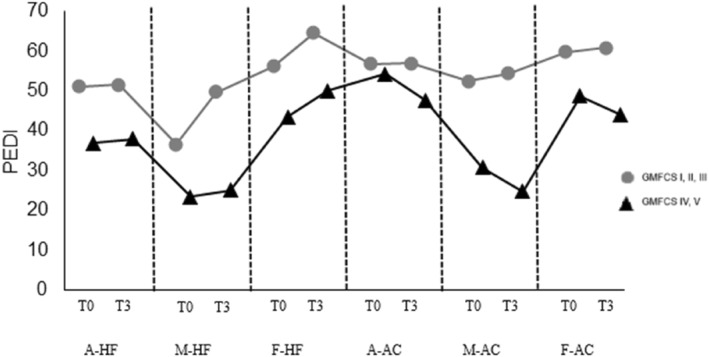
PEDI relationship according to GMFCS levels. A, self‐care; AC, caregiver assistance; F, social function; HF, functional skills; M, mobility. T0 (assessment) and T3 (last reassessment) in each functional skill and caregiver assistance.

The relationship between PEDI and GMFCS levels was analyzed at T0 and T3 as a measure of functionality (Table 4 and Figure 2). Children were analyzed according to their age group. In the functional skills item (mobility), children with levels I, II and III showed a significant improvement from T0 to T3 (*p* 0.001). In terms of social function, levels I, II and III also showed a significant improvement (*p* 0.030). Children in levels IV and V did not show improvement in the functional skills item (self‐care, mobility and social function) nor caregiver assistance.

**TABLE 4:** PEDI relationship according to GMFCS.

	GMFCS	T0	T3	*p* value
PEDI self‐care HF	I, II and III	51.1 ± 32.5	51.6 ± 39.7	0.89
	IV and V	36.9 ± 15.4	37.9 ± 24.9	0.872
HF mobility	I, II and III	36.7 ± 26.1	49.6 ± 34.2	**0.001** *
	IV and V	23.5 ± 10.1	25 ± 15.2	0.603
Social function HF	I, II and III	56.2 ± 24.3	64.5 ± 31	**0.03** *
	IV and V	43.5 ± 28.8	50 ± 35.3	0.124
PEDI self‐care AC	I, II and III	56.8 ± 32.8	57 ± 35	0.899
	IV and V	54.2 ± 21.4	47.7 ± 13.1	0.364
AC mobility	I, II and III	52.4 ± 39.6	54.4 ± 40	0.13
	IV and V	30.7 ± 15.5	24.8 ± 13	0.369
Social function AC	I, II and III	59.8 ± 39.3	60.7 ± 39.7	0.303
	IV and V	48.6 ± 17.2	44.1 ± 20.7	0.28

*Note:* Data are presented as mean ± SD (mean and standard deviation). Means that do not share letters differ from each other. Sidak test for multiple comparisons.

Abbreviations: GMFCS, gross motor function classification system. PEDI HF, functional skills. PEDI AC, caregiver assistance.

^*^ *p*‐value < 0.05.

## 4 Discussion

This longitudinal study presents the characterization of children with CP, analyzing the relationship between gross motor function and performance in activities of daily living, mobility and social function. The analysis of the multimodal neurorehabilitation program showed that gross motor function, measured by the GMFM‐66, improved for both groups in the total score. Groups IV and V improved only in dimension B (sitting), while groups I, II, and III showed significant changes in dimensions D (standing) and E (walking, running, and jumping). Regarding mobility and social function, assessed by PEDI—Part I, only levels I, II, and III of the GMFCS showed significant improvement. These findings reflect the relevance of neurorehabilitation in the development of these children.

Categorization of children into GMFCS groups revealed distinct patterns of motor impairment. Children in levels IV and V predominantly presented quadriparesis, whereas those in levels I, II and III presented hemiparesis. Thus, most of children used these devices and were totally dependent. In addition, greater spasticity in the hamstring muscles was observed in these groups, reinforcing previous findings on motor patterns in CP (Zardo et al. 2022). The severity of motor impairment justifies the lower functional evolution in the most affected groups, who face significant challenges in activities such as transfers and functional mobility. In contrast, children in levels I, II and III have a lower functional impact (Saleh et al. 2019), which explains their greater evolution in gross motor function and functional skills throughout the study.

Adapting to the environment and using assistive devices are essential to promote greater functional independence in children severely impaired (Burgess et al. 2021; Lidbeck et al. 2023). For levels IV and V, providing the experience of walking can be a relevant goal for inclusion and well‐being (Jackman et al. 2022), while at milder levels, advances in mobility directly impact participation in daily activities.

The differences between the groups were reflected in the progress observed. Children in levels I, II and III made progress in dimensions D (standing) and E (walking, running and jumping) of the GMFM‐66, while in levels IV and V the main progress occurred in dimension B (sitting), which is essential for children who remain in this position for long periods and have difficulty in postural adjustments (Carlberg and Hadders‐Algra 2005). Acquiring the skills of sitting and walking is crucial for the independence and quality of life. However, this control develops differently in children with CP, becoming a challenge for motor skills such as locomotion (Vasani et al. 2025).

Progress in self‐care, mobility and social function was more evident in children with levels I, II and III, with significant differences in mobility and social function. In levels IV and V, the changes were not statistically significant, but the level of assistance remained unchanged, indicating a stabilization in the level of caregiver assistance. Mobility is directly related to independence in daily activities, reinforcing the need for realistic therapeutic plans for this population (Kim et al. 2017), considering social participation and recreational activities, which are essential for functional development. In the present study, 94.4% of children in levels I, II and III and 75% of children in levels IV and V participated in recreational activities, highlighting the need to encourage social interaction and promote environmental adaptations that favor the well‐being of these children (Sousa Junior et al. 2023).

Regarding neurorehabilitation, all children received NDT‐based intervention and 50% performed locomotor training on a treadmill. NDT is widely used to improve gross motor function, improve postural control and facilitate muscle activity (Zanon et al. 2019; Furtado et al. 2022; Raipure et al. 2023). Locomotor training on a treadmill can help increase walking speed and improve transfer skills (Jackman et al. 2022). Other methods used included suit therapy, which combines a therapeutic suit with intensive exercises (Neves et al. 2013; Da Silva Júnior 2018). The investigation of different therapeutic approaches reinforces the importance of a diversified clinical practice, as in a single session, therapists can use different methods to achieve their objective (Neves et al. 2013; Camara Machado et al. 2017; Da Silva Júnior 2018; Raipure et al. 2023).

The findings of this study are partially aligned with the literature indicating that neurorehabilitation‐based interventions can promote functional gains in children with cerebral palsy. Previous studies show that Neurodevelopmental Treatment (NDT) can improve specific components of gross motor function, particularly those related to proximal postural control and transitional movements (Palisano et al. 1997; Novak et al. 2013). For children classified as GMFCS levels I–III, previous reports suggest meaningful gains primarily in GMFM‐66 Dimensions D (standing) and E (walking), which are consistent with the improvements observed in our sample.

Studies have demonstrated a significant relationship between motor severity (GMFCS levels IV–V) and trunk control during sitting, suggesting that maintaining or improving this skill is a realistic functional goal for children with higher levels of impairment. However, controlled longitudinal studies documenting consistent progress in specific GMFM dimensions for levels IV–V remain scarce (Montero et al. 2015). Our findings reflect similar patterns: children with levels IV–V showed improvements primarily in sitting stability (GMFM—Dimension B).

Existing studies still show considerable heterogeneity in protocols and a lack of methodological standardization (Loffi et al. 2024). In this context, our results suggest that the structured application of multiple interventions within a neurologically focused rehabilitation framework may expand opportunities for motor practice and support learning. However, this hypothesis requires confirmation through rigorously controlled clinical trials.

A review of longitudinal studies in neurorehabilitation reveals substantial variation in intervention duration, ranging from 4‐week and 8‐week protocols to follow‐ups extending up to 1 year (Ho et al. 2017; Kim et al. 2017; Raipure et al. 2023; Vasani et al. 2025). This methodological heterogeneity limits direct comparison and emphasizes the need for studies with more consistent designs and sufficiently long monitoring periods to detect meaningful functional changes in children with cerebral palsy.

Despite this variability, few longitudinal studies incorporate multimodal protocols adjusted to GMFCS levels with functional monitoring using standardized tools such as GMFM‐66 and PEDI, underscoring the relevance of the present findings.

This study presents several limitations that should be considered when interpreting the results. The sample was selected by convenience and composed of children already participating in continuous rehabilitation, which may introduce selection bias. The absence of a control group limits causal inference regarding the observed improvements. Although the 9‐month follow‐up provides relevant longitudinal insight, longer monitoring may better capture the sustainability of functional gains.

Nevertheless, the relevance of this study remains, as children were observed in their real‐life therapeutic routines, enhancing ecological validity and reflecting everyday clinical practice.

Another limitation relates to the heterogeneity of complementary therapies received by participants. Although non‐evidence‐based interventions were excluded from analyses, their presence in the rehabilitation environment may generate indirect effects that are challenging to measure. Future research should adopt controlled designs, rigorous dose standardization, and extended longitudinal evaluations to strengthen evidence on combined interventions in CP.

The combination of essential neurorehabilitation approaches demonstrated the potential to promote functional gains in children with cerebral palsy over a 9‐month follow‐up period. Among children classified as GMFCS levels I–III, the main advances occurred in GMFM‐66 Dimensions D (standing) and E (walking), indicating improvements in functional mobility and overall motor performance. For participants in GMFCS levels IV–V, progress was predominantly observed in Dimension B (sitting), reflecting improved postural stability and trunk control.

These findings reinforce that combined interventions, when structured within a standardized clinical protocol and tailored to the functional profile of each GMFCS level, may contribute meaningfully to motor development in children with CP.

## 5 Implications for Physical Therapy Practice

Neurorehabilitation plays an important role in improving gross motor function in children diagnosed with cerebral palsy, significantly impacting performance in self‐care activities, mobility, and social function.

Improving gross motor function can promote significant advances in children classified as GMFCS levels I, II, and III. During activities such as standing, walking, running, and jumping, physical therapy intervention contributes to the development and improvement of motor coordination, muscle strength, and balance, among others. For children with greater motor impairment, it is essential to stabilize their sitting posture. This improvement allows for better postural control, facilitating the performance of daily activities and promoting greater comfort and social interaction.

In addition to motor improvements, neurorehabilitation and long‐term monitoring also favor the mobility and social participation of children with cerebral palsy at GMFCS levels I, II, and III. Greater independence in locomotion allows for greater integration into school and social environments, promoting emotional and psychological well‐being. In the most severe cases (GMFCS levels IV and V), it contributes to maintaining and stabilizing the level of care required by caregivers. This means that, even when faced with significant motor challenges, the child can achieve a level of functionality that reduces caregiver burden and improves the quality of care provided.

The use of a Multimodal Neurorehabilitation Program has shown benefits for children at all GMFCS levels. Evidence‐based interventions promote better functional outcomes, making the physiotherapy approach more effective and adapted to the individual needs of each child.

Therefore, physiotherapy, through neurorehabilitation, represents a crucial intervention in promoting functional independence, activity and participation for children with cerebral palsy, reinforcing the importance of individualized and continuous treatment.

## Author Contributions

**Franciele Zardo:** conceptualization, writing – original draft, formal analysis, writing – review and editing. **Daiane Bridi:** data collection. **Monique Schorn:** data collection. **Isabelle Cousseau:** data collection. **Ketlyn Leal:** data collection. **Sabrina Knop:** data collection. **Fernanda Cechetti:** review and editing.

## Funding

The authors have nothing to report.

## Disclosure

We confirm that this manuscript has not and is not under consideration for publication elsewhere. The views expressed are those of the authors and not necessarily those of the NHS, the NIHR, or the Department of Health and Social Care.

## Ethics Statement

This is a prospective, longitudinal cohort study approved by the Ethics Committee of the Universidade Federal de Ciências da Saúde de Porto Alegre (No. 5.040.891; CAAE 50820221.9.0000.5345). ClinicalTrials.gov registration ID NCT05608954.

## Consent

Informed consent was obtained from all participants.

## Conflicts of Interest

The authors declare no conflicts of interest.

## Data Availability

The data used for this study are available from the corresponding author upon reasonable request.

## References

[pri70158-bib-0001] Berker, A. N. , and M. S. Yalçin . 2008. “Cerebral Palsy: Orthopedic Aspects and Rehabilitation.” Pediatric Clinics of North America 55, no. 5: 1209–ix. 10.1016/j.pcl.2008.07.011. 18929061

[pri70158-bib-0002] Burgess, A. , R. N. Boyd , M. D. Chatfield , J. Ziviani , J. Wotherspoon , and L. Sakzewski . 2021. “Função da mão e Autocuidado em Crianças Com Paralisia Cerebral.” Medicina do desenvolvimento e neurologia infantil 63, no. 5: 576–583. 10.1111/dmcn.14783. 33354794

[pri70158-bib-0003] Burgess, A. , S. Reedman , M. D. Chatfield , R. S. Ware , L. Sakzewski , and R. N. Boyd . 2022. “Development of Gross Motor Capacity and Mobility Performance in Children With Cerebral Palsy: A Longitudinal Study.” Developmental Medicine and Child Neurology 64, no. 5: 578–585. 10.1111/dmcn.15112. 34800033

[pri70158-bib-0004] Camara Machado, F. R. , P. P. Antunes , J. M. Souza , A. C. D. Santos , D. C. Levandowski , and A. A. Oliveira . 2017. “Melhoria Motora Usando Dispositivos de Jogo com Detecção de Movimento para Reabilitação De Paralisia Cerebral.” Jornal de comportamento motor 49, no. 3: 273–280. 10.1080/00222895.2016.1191422. 27593342

[pri70158-bib-0005] Carlberg, E. B. , and M. Hadders‐Algra . 2005. “Postural Dysfunction in Children With Cerebral Palsy: Some Implications for Therapeutic Guidance.” Neural Plasticity 12, no. 2–3: 221–272. 10.1155/NP.2005.221. 16097490 PMC2565463

[pri70158-bib-0006] Chagas, P. S. C. , A. G. Lemos , K. M. A. Ayupe , et al. 2024. “Functioning Profile and Related Impairments of Children and Adolescents With Cerebral Palsy—Participa Brazil Preliminary Results.” BMC Pediatrics 24, no. 1: 719. 10.1186/s12887-024-05210-2. 39529069 PMC11552143

[pri70158-bib-0007] Chagas, P. S. C. , E. D. D. Magalhães , R. R. Sousa Junior , et al. 2023. “Desenvolvimento De Crianças, Adolescentes E Jovens Adultos Com Paralisia Cerebral De Acordo Com a CIF? Uma Revisão Do Escopo.” Developmental Medicine and Child Neurology 65, no. 6: e61–e69. 10.1111/dmcn.15487. 36529898

[pri70158-bib-0008] Colver, A. , C. Fairhurst , and P. O. Pharoah . 2014. “Paralisia Cerebral.” Lancet (Londres, Inglaterra) 383, no. 9924: 1240–1249. 10.1016/S0140-6736(13)61835-8. 24268104

[pri70158-bib-0009] Da Silva Júnior, R. A. 2018. “Avaliação Da Função Motora grossa em Pacientes Com Encefalopatia Crônica Não Progressiva Da Infância Com O Uso Da Suit Terapia.” Supplement, Fisioterapia Brasil 19, no. 5: S33–S42. 10.33233/fb.v19i5.2596.

[pri70158-bib-0010] De Mello Sposito, M. M. , and M. Riberto . 2010. “Avaliação Da Funcionalidade Da Criança Com Paralisia Cerebral Espástica.” Revista Acta Fisiátrica 17, no. 2: 50–61. 10.11606/issn.2317-0190.v17i2a103312.

[pri70158-bib-0011] Eliasson, A. C. , L. Nordstrand , M. Backheden , and M. Holmefur . 2023. “Longitudinal Development of Hand Use in Children With Unilateral Spastic Cerebral Palsy From 18 Months to 18 Years.” Developmental Medicine and Child Neurology 65, no. 3: 376–384. 10.1111/dmcn.15370. 35899928 PMC10087588

[pri70158-bib-0012] Feldman, A. B. , S. M. Haley , and J. Coryell . 1990. “Concurrent and Construct Validity of the Pediatric Evaluation of Disability Inventory.” Physical Therapy 70, no. 10: 602–610. 10.1093/ptj/70.10.602. 2217539

[pri70158-bib-0013] Furtado, M. A. S. , K. M. A. Ayupe , I. S. Christovão , et al. 2022. “Fisioterapia em Crianças Com Paralisia Cerebral No Brasil: Uma Revisão De Escopo.” Medicina do desenvolvimento e neurologia infantil 64, no. 5: e2–e12. 10.1111/dmcn.15094. 34689323

[pri70158-bib-0014] Haley, S. M. , W. I. Coster , Y. C. Kao , et al. 2010. “Lessons From Use of the Pediatric Evaluation of Disability Inventory: Where Do We Go From Here?” Pediatric Physical Therapy: The official publication of the Section on Pediatrics of the American Physical Therapy Association 22, no. 1: 69–75. 10.1097/PEP.0b013e3181cbfbf6. PMC3631526 20142708

[pri70158-bib-0015] Harb, A. , and S. Kishner . 2023. “Modified Ashworth Scale.” In Statpearls. StatPearls Publishing. 32119459

[pri70158-bib-0016] Ho, P. C. , C. H. Chang , M. Granlund , and A. W. Hwang . 2017. “The Relationships Between Capacity and Performance in Youths With Cerebral Palsy Differ for GMFCS Levels.” Pediatric Physical Therapy: The official publication of the Section on Pediatrics of the American Physical Therapy Association 29, no. 1: 23–29. 10.1097/PEP.0000000000000332. 27984462

[pri70158-bib-0017] Jackman, M. , L. Sakzewski , C. Morgan , et al. 2022. “Interventions to Improve Physical Function for Children and Young People With Cerebral Palsy: International Clinical Practice Guideline.” Developmental Medicine and Child Neurology 64, no. 5: 536–549. 10.1111/dmcn.15055. 34549424

[pri70158-bib-0018] Kim, K. , J. Y. Kang , and D. H. Jang . 2017. “Relationship Between Mobility and Self‐Care Activity in Children With Cerebral Palsy.” Annals of rehabilitation medicine 41, no. 2: 266–272. 10.5535/arm.2017.41.2.266. 28503460 PMC5426278

[pri70158-bib-0019] Kollen, B. J. , S. Lennon , B. Lyons , et al. 2009. “The Effectiveness of the Bobath Concept in Stroke Rehabilitation: What Is the Evidence?” Stroke 40, no. 4: e89–e97. 10.1161/STROKEAHA.108.533828. 19182079

[pri70158-bib-0020] Lidbeck, C. , H. Häbel , C. Martinsson , K. Pettersson , and K. Löwing . 2023. “Motor Development in Children With Cerebral Palsy in Sweden‐A Population‐Based Longitudinal Register Study.” Children 10, no. 12: 1864. 10.3390/children10121864. 38136066 PMC10741609

[pri70158-bib-0021] Loffi, R. G. , D. O. Souto , T. K. F. Cruz , et al. 2024. “Narrative Review of the Theoretical–Methodological Foundations of the TREINI Program.” Children 11, no. 10: 1181. 10.3390/children11101181. 39457146 PMC11505838

[pri70158-bib-0022] Meseguer‐Henarejos, A. B. , J. Sánchez‐Meca , J. A. López‐Pina , and R. Carles‐Hernández . 2018. “Inter‐ and Intra‐Rater Reliability of the Modified Ashworth Scale: A Systematic Review and Meta‐Analysis.” European Journal of Physical and Rehabilitation Medicine 54, no. 4: 576–590. 10.23736/S1973-9087.17.04796-7. 28901119

[pri70158-bib-0023] Montero, M. S. , A. Gómez‐Conesa , and M. D. Hidalgo Montesinos . 2015. “Associação Entre Função Motora Grossa E Controle Postural em Sentar em Crianças Com Paralisia Cerebral: Um Estudo Correlacional Na Espanha.” Pediatra BMC 15 (September): 124 PMID: 26376627; PMCID: PMC4571109. 10.1186/s12887-015-0442-4.

[pri70158-bib-0024] Morgan, C. , J. Darrah , A. M. Gordon , et al. 2016. “Effectiveness of Motor Interventions in Infants With Cerebral Palsy: A Systematic Review.” Developmental Medicine and Child Neurology 58, no. 9: 900–909. 10.1111/dmcn.13105. 27027732

[pri70158-bib-0025] Neves, E. B. , E. Krueger , M. C. Stéphani de Pol , N. De Oliveira , A. F. Szinke , and M. de Oliveira Rosário . 2013. “Benefícios da Terapia Neuromotora Intensiva (TNMI) Para o Controle Do tronco de Crianças Com Paralisia Cerebral.” Rev Neurocienc 21, no. 4: 549–555. 10.4181/RNC.2013.21.876.7p.

[pri70158-bib-0026] Novak, I. , S. McIntyre , C. Morgan , et al. 2013. “A Systematic Review of Interventions for Children With Cerebral Palsy: State of the Evidence.” Developmental Medicine and Child Neurology 55, no. 10: 885–910. 10.1111/dmcn.12246. 23962350

[pri70158-bib-0027] Novak, I. , C. Morgan , M. Fahey , et al. 2020. “State of the Evidence Traffic Lights 2019: Systematic Review of Interventions for Preventing and Treating Children With Cerebral Palsy.” Current neurology and neuroscience reports 20, no. 2: 3. 10.1007/s11910-020-1022-z. 32086598 PMC7035308

[pri70158-bib-0028] Palisano, R. , P. Rosenbaum , S. Walter , D. Russell , E. Wood , and B. Galuppi . 1997. “Development and Reliability of a System to Classify Gross Motor Function in Children With Cerebral Palsy.” Developmental Medicine and Child Neurology 39, no. 4: 214–223. 10.1111/j.1469-8749.1997.tb07414.x. 9183258

[pri70158-bib-0029] Raipure, A. , R. K. Kovela , and P. Harjpal . 2023. “Effectiveness of Neurodevelopmental Treatment and Sensory Integration Therapy on Gross Motor Function, Balance and Gait Parameters in Children With Spastic Diplegia.” Cureus 15, no. 8: e43876. 10.7759/cureus.43876. 37746405 PMC10511346

[pri70158-bib-0030] Rosenbaum, P. , N. Paneth , A. Leviton , et al. 2007. “A Report: The Definition and Classification of Cerebral Palsy April 2006. Dev Med Child Neurol Suppl. 2007 Feb;109:8‐14. Erratum in.” Developmental Medicine and Child Neurology, (June) 49, no. 6: 480: PMID: 17370477. 17370477

[pri70158-bib-0031] Russell, D. J. , L. M. Avery , P. L. Rosenbaum , P. S. Raina , S. D. Walter , and R. J. Palisano . 2000. “Improved Scaling of the Gross Motor Function Measure for Children With Cerebral Palsy: Evidence of Reliability and Validity.” Physical Therapy 80, no. 9: 873–885. 10.1093/ptj/80.9.873. 10960935

[pri70158-bib-0032] Saleh, M. , N. A. Almasri , S. H. Malkawi , and S. Abu‐Dahab . 2019. “Associations Between Impairments and Activity Limitations Components of the International Classification of Functioning and the Gross Motor Function and Subtypes of Children With Cerebral Palsy.” Journal of Physical Therapy Science 31, no. 4: 299–305. 10.1589/jpts.31.299. 31036999 PMC6451943

[pri70158-bib-0033] Sousa Junior, R. R. , D. O. Souto , A. C. R. Camargos , G. L. Clutterbuck , and H. R. Leite . 2023. “Moving Together Is Better: A Systematic Review With Meta‐Analysis of Sports‐Focused Interventions Aiming to Improve Physical Activity Participation in Children and Adolescents With Cerebral Palsy.” Disability & Rehabilitation 45, no. 15: 2398–2408. 10.1080/09638288.2022.2098394. 35853235

[pri70158-bib-0034] Vasani, P. , A. Narayan , A. Nayak , M. Alsulaimani , and A. R. Alzahrani . 2025. “Anticipatory and Compensatory Postural Adjustments in Sitting and Standing Positions During Functional Activities in Children With Cerebral Palsy.” Physiotherapy Research International: the journal for researchers and clinicians in physical therapy 30, no. 1: e70028. 10.1002/pri.70028. 39804176 PMC11727814

[pri70158-bib-0035] Zanon, M. A. , R. L. Pacheco , C. O. C. Latorraca , A. L. C. Martimbianco , D. V. Pachito , and R. Riera . 2019. “Neurodevelopmental Treatment (Bobath) for Children With Cerebral Palsy: A Systematic Review.” Journal of Child Neurology 34, no. 11: 679–686. 10.1177/0883073819852237. 31179823

[pri70158-bib-0036] Zardo, F. , T. Paludo , B. T. P. D. Mattos , B. Frata , C. C. Ling , and F. Cechetti . 2022. “Analysis of Muscle Activation in Children and Adolescents With Severe Cerebral Palsy.” Fisioterapia em Movimento 35: e35115. 10.1590/fm.2022.35115.

